# Associations between semi-quantitative evaluation of intracranial arterial calcification and total cerebral small vessel disease burden score: a retrospective case-control study

**DOI:** 10.3389/fneur.2024.1417186

**Published:** 2024-07-31

**Authors:** Peng Chen, Tiejun Liu, Yin Wei, Zhen Ma, Tao Lu, Suxi Lan, Jinling Xie, Shen Mo

**Affiliations:** ^1^Department of Radiology, Guangxi International Zhuang Medicine Hospital, Nanning, China; ^2^Department of Ultrasound, Guangxi International Zhuang Medicine Hospital, Nanning, China

**Keywords:** cerebral small vessel disease, intracranial arterial calcification, magnetic resonance imaging, computed tomography, white matter hyperintensities

## Abstract

**Background and purpose:**

Arteriosclerotic cerebral small vessel disease (aCSVD) is a cause of cognitive impairment, dementia, and stroke. Developing a better understanding of the risk factor of aCSVD is key to reducing the incidence of these conditions. This study investigated the association between intracranial arterial calcification (IAC) and total cerebral small vessel disease (CSVD) burden score.

**Materials and methods:**

This is a retrospective study, the subjects were transient ischemic attack (TIA) or acute ischemic stroke (AIS) patients. The data of 303 inpatients admitted to our study hospital between December 2018 and July 2020 were analyzed. Four imaging markers of CSVD (lacunes, white matter hyperintensities, cerebral microbleeds, and enlarged perivascular spaces) were evaluated by magnetic resonance imaging, and a total CSVD burden score was calculated. The experimental group was divided into four subgroups according to total CSVD burden score (1–4 points). Patients without CSVD (0 points) served as the control group. Head computerized tomography (CT) scans were used to assess ICA, using Babiarz’s method. The correlations between IAC and single imaging markers of CSVD were determined using Spearman’s rank correlation. Binary logic regression analysis and multivariate ordered logic regression analysis were used to determine the associations between IAC and aCSVD.

**Results:**

IAC was positively correlated with total CSVD burden score (*r* = 0.681), deep white matter hyperintensities (*r* = 0.539), periventricular white matter hyperintensities (*r* = 0.570), cerebral microbleeds (*r* = 0.479), lacunes (*r* = 0.541), and enlarged perivascular spaces (*r* = 0.554) (all *p* < 0.001). After adjusting for the confounding factors of age, diabetes, and hypertension, aCSVD was independently associated with IAC grade 1–2 [odds ratio (OR) = 23.747, 95% confidence interval (CI) = 8.376–67.327] and IAC grade 3–4 (OR = 30.166, 95% CI = 8.295–109.701). aCSVD severity was independently associated with IAC grade 3–4 (OR = 4.697, 95% CI = 1.349–16.346).

**Conclusion:**

IAC is associated with the total CSVD burden score and single imaging signs.

## 1 Introduction

Cerebral small vascular disease (CSVD) is a series of clinical, imaging, and pathological syndromes commonly caused by structural and functional changes in the small arteries, arterioles, capillaries, and venules, arteriosclerotic CSVD (aCSVD) is one of the major subtypes of the CSVD spectrum (1). There are six closely correlated magnetic resonance imaging (MRI) markers of CSVD, including recent small subcortical infarct, white matter hyperintensities (WMHs), lacunes, cerebral microbleeds (CMBs), enlarged perivascular spaces (EPVS), and atrophy (2). Chronic CSVD can cause cognitive function decline (3), depression (4), and gait disturbance (5). CSVD is responsible for approximately one-quarter of ischemic and hemorrhagic strokes (6). It is also the most common cause of vascular dementia, which is often associated with Alzheimer’s disease and leads to further deterioration in cognitive function (6–8). The common risk factors for CSVD are hypertension, aging, smoking, obesity (9–11), which eventually cause the vascular glio-neuronal unit injury through various ways (12). Cerebral amyloid angiopathemia is also a common cause of CSVD in the elderly, and is associated with the persistent deposition of amyloid b-peptide in the wall of the arteriole. It is also a common cause of other neurodegenerative diseases, such as Alzheimer’s disease (13). Therefore, identifying patients at risk of CSVD is integral to reducing the burden of stroke, dementia, and dyskinesia. Some studies have shown that intracranial arterial calcification (IAC) is a risk factor for CSVD, while other studies have not found any associations between IAC and CMBs and EPVSs (14, 15). It is of great value to clarify the relationship between them, because CSVD is a disease caused by cerebral microvascular diseases, and to clarify the relationship between macroangiopathy and CSVD may provide a new direction for further study of its pathogenesis. Most existing research on the topic investigated the associations between single MRI markers of CSVD and IAC (14–18). To our knowledge, no study has combined these markers into an overall measure of CSVD burden when investigating the association between CSVD and IAC. A measure of the overall burden of CSVD better reflects the total effects of CSVD on brain tissue than one or two markers of CSVD alone (19). Therefore, to further understand the risk factors of aCSVD, this study aimed to explore the associations between the overall burden of aCSVD and IAC.

## 2 Materials and methods

### 2.1 Clinical data

This was a retrospective case-control study of patients admitted to Hospital between December 2018 and July 2020 with a transient ischemic attack (TIA) or acute ischemic stroke (AIS). All patients presented to the study hospital within 7 days of symptom onset and underwent computed tomography (CT) and MRI examination during the inpatient stay. Ethics approval was obtained from the study hospital’s Medical Ethics Committee (ethics approval number: [2021]-006).

The case group consisted of patients with aCSVD, as determined by the presence of one or more radiological markers of CSVD on MRI (2), including WMHs, lacunes, CMBs, and EPVSs. Excluded from the case group were patients with: (1) other possible sources of white matter hypoattenuation identified through a chart review – such as multiple sclerosis, toxic encephalopathy, acute disseminated encephalomyelitis, vasculitis, metabolic diseases, and hydrocephalus; (2) intracranial vascular malformation, history of head trauma, large area cerebral infarction (diffusion weighted image lesions larger than one-third of the territory of the middle cerebral artery), or tumor; (3) poor quality brain MRI or CT images (taken during the inpatient stay) that could not be evaluated; and (4) non-arteriosclerotic CSVD, such as inherited CSVD or probable cerebral amyloid angiopathy (CAA). According to the total CSVD burden score proposed by Staals (19), the case group was categorized into four subgroups (total CSVD burden score 1–4).

The control group comprised patients without CSVD. Clinical data of interest included age, sex, hypertension, diabetes, hyperlipidemia, atrial fibrillation, coronary heart disease, smoking and other cerebrovascular disease risk factors (all obtained from hospital medical records).

### 2.2 Neuroimaging

Brain MRIs were performed with a GE Discovery MR750W 3.0 T scanner mute MRI with a 19-channel receive array. The MRI sequence included: T1WI, T2WI, fluid-attenuated inversion recovery (FLAIR), and susceptibility weighted imaging (SWI). Specific parameters included: axial T2WI:TR 6,000 ms, TE 102 ms, FOV 240 mm × 240 mm, slice thickness 5 mm, slice gap 1 mm. Axial T1WI:TR 1,750 ms, TE 24 ms, FOV 240 mm × 240 mm, slice thickness 5 mm, slice gap 1 mm. Axial FLAIR:TR 9,000 ms, TE 120 ms, inversion time (TI) 2,474 ms, FOV 240 mm × 240 mm, slice thickness 5 mm, slice gap 1 mm. Axial SWI:TR = 39 ms, TE = 24 ms, flip angle 15°, layer number 50, slice thickness 3 mm, excitation times 1, FOV 240 mm × 180 mm, matrix 320 × 256. Axial DWI:TR 5,822 ms, TE 80 ms, FOV 240 mm × 240 mm, slice thickness 5 mm, slice gap 1 mm. Head CTs were performed with a GE Revolution 256 row spiral CT, slice thickness 3 mm, slice gap 3 mm FOV 512 × 512. The scanning range was from the inferior edge of the external ear foramen to the top of the skull.

### 2.3 Image analysis methods

CT and MRI images were evaluated by two neuroradiologists with 11 and 13 years of diagnostic imaging experience. Before evaluating study images, they received standardized training on study protocols. The two readers independently evaluated CT and MRI images displayed on a PACS display. Measures of inter-observer reliability displayed high inter-rater consistency, wherein the kappa values for the presence of deep WMHs, periventricular WMHs, lacunes, EPVSs, CMBs, total CSVD burden score, and IAC grade were 0.885, 0.896, 0.941, 0.868, 0.875, 0.921, and 0.921, respectively (all *p* < 0.05). Any disagreements were resolved through discussion between the two readers.

**Figure 1:**
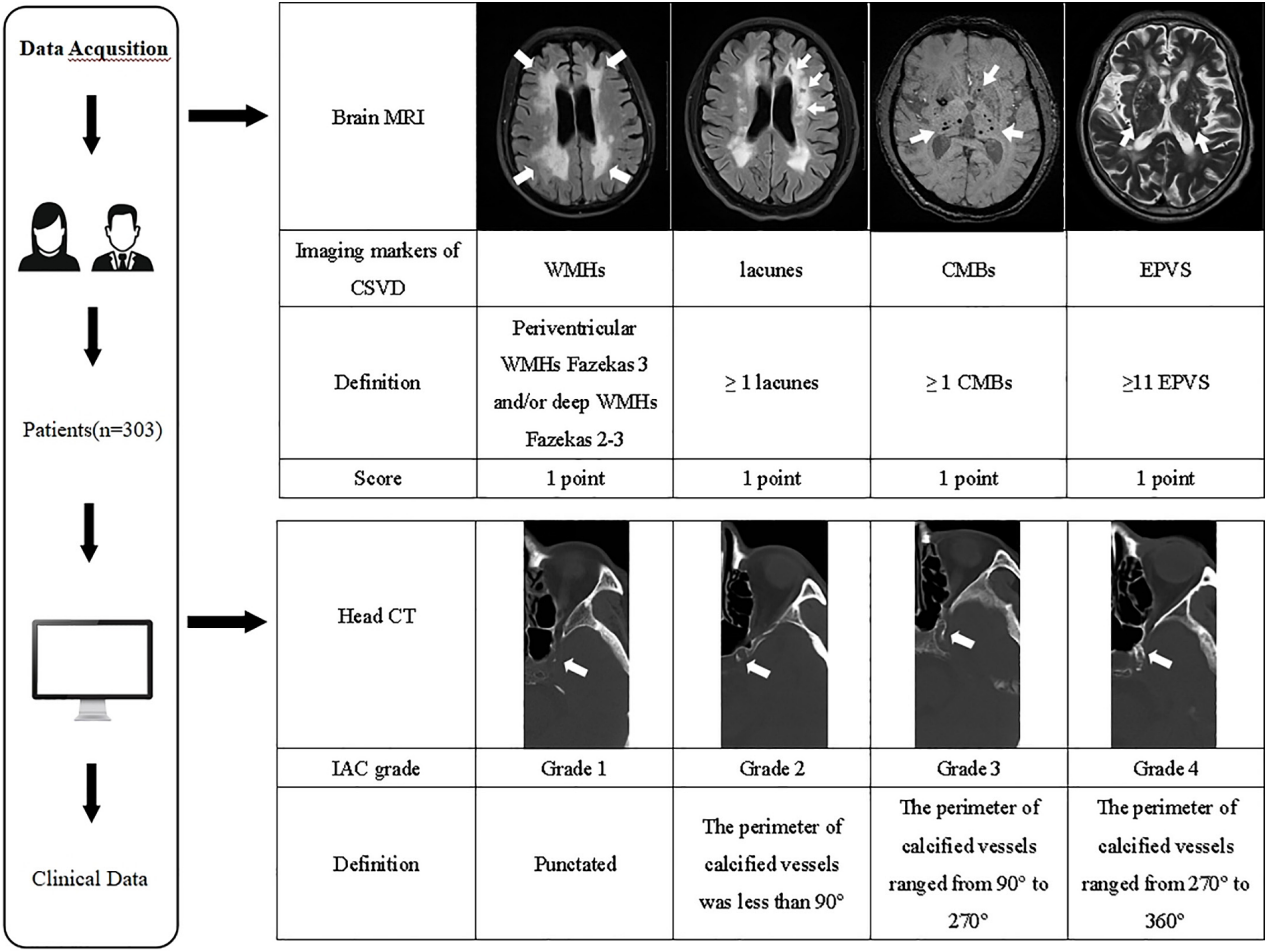
The imaging evaluation flowchart of IAC and CSVD. MRI, magnetic resonance imaging; CSVD, cerebral small vessel disease; WMHs, white matter hyperintensities; CMBs, cerebral microbleeds; EPVS, enlarged perivascular spaces; CT, computed tomography; IAC, intracranial arterial calcification.

The imaging evaluation of IAC and CSVD is shown in Figure 1.

Readers use the Babiarz method (20) to evaluate IAC on head CT images. Fixed bone window settings (window 800 HU, window width 2,000 HU), slice thickness (3 mm), and slice gap (3 mm) were used. IAC evaluation criteria included observing the internal carotid artery’s cavernous sinus section and evaluating the vessels with the highest calcification grade. Scores were assigned as follows: (1) Grade 1: punctated; (2) Grade 2: the perimeter of calcified vessels was less than 90°; (3) Grade 3: the perimeter of calcified vessels ranged from 90° to 270°; (4) Grade 4: the perimeter of calcified vessels ranged from 270° to 360° (Figure 1).

The total CSVD burden score was calculated from brain MRIs (19), with a score range of 0–4. The specific criteria were: (1) lacunes: 1 point when ≥1 lacunes; (2) CMBs: 1 point when ≥1 CMBs; (3) EPVSs: 1 point when basal ganglia lesions were 2–4 (21); (4) WMHs: 1 point when deep WMHs were assessed as Fazekas grade 2–3 and/or periventricular WMHs were assessed as Fazekas grade 3. The sum of these four criteria was the total CSVD burden score (Figure 1).

### 2.4 Statistical analysis

Statistical analyses were carried out using SPSS 21.0 software. Cohen’s kappa was used to evaluate inter-observer reliability levels between both readers for deep WMHs, periventricular WMHs, lacunes, EPVSs, CMBs, total CSVD burden score, and IAC grade. Normally distributed data are presented as means and standard deviations ( $\bar{x}\pm s$ ). Non-normally distributed data are presented as medians and 25th and 75th percentiles (M, P25 and P75). Counts are presented as *n* (%). A Chi-square test or Kruskal–Wallis test was used to compare data between the five groups, and Spearman’s rank correlation was used to measure associations between total CSVD burden score, single markers of CSVD, and IAC. Lastly, binary logistic regression was carried out with CSVD (≥1 point) as the dependent variable, while multiple-ordered logic regression analysis was carried out with CSVD severity (1–4 points) as the dependent variable. *p* < 0.05 was considered to be statistically significant.

## 3 Results

**Figure 2:**
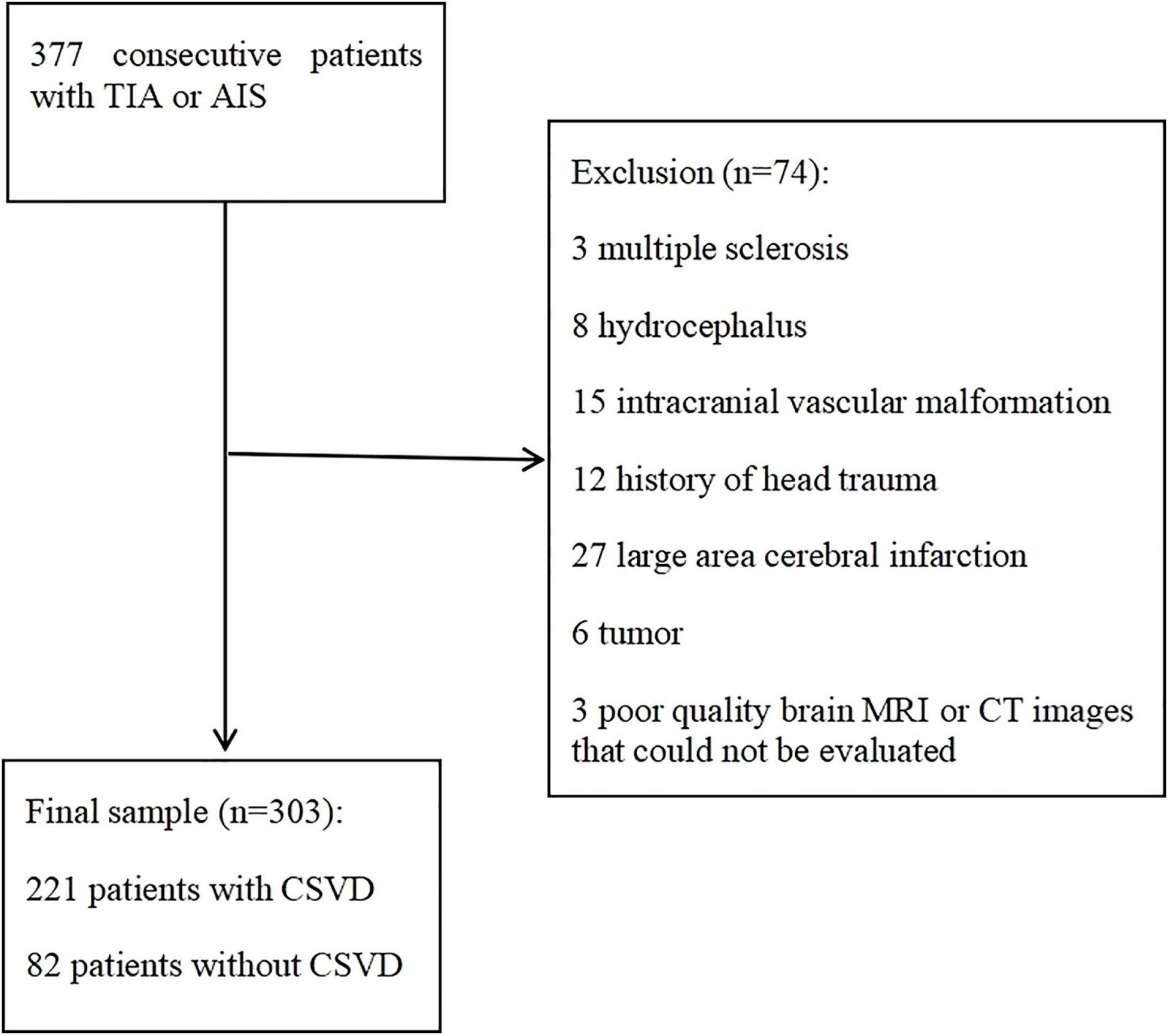
Flowchart of patients selection. AIS, acute ischemic stroke; TIA, transient ischemic attack; CT, computed tomography; MRI, magnetic resonance imaging.

Three hundred thirty-seven consecutive patients admitted with TIA or AIS were screened and 303 met inclusion criteria (Figure 2). Patients included in the study were aged 16–90 years (mean = 58.01 ± 14.77 years). 205 (67.7%) were male and 98 (32.3%) were female. There are 122 patients with TIA and 181 patients with AIS. Regarding the distribution of lesions in AIS patients, there are 62 cases (34.3%) in the left hemisphere, 55 cases (30.4%) in the right hemisphere, 11 cases (6.1%) in the bilateral hemisphere, 37 cases (20.4%) in the brain stem and 16 cases (8.8%) in the cerebellum. 221 (72.9%) patients comprised the case group (aCSVD), of which 207 (93.7%) had IAC. The distribution of patients with aCSVD across total CSVD burden score categories was uniform, with 57 (18.8%), 59 (19.5%), 53 (17.5%), and 52 (17.2%) of patients with total CSVD burden score 1, 2, 3, and 4, respectively. 82 (27.1%) patients were in the control group (no CSVD), of which 14 (17.1%) had IAC.

### 3.1 Comparison of clinical data’s between the five groups

As detailed in Table 1, comparisons of clinical data between the five groups (no CSVD, CSVD scores 1–4) revealed statistically significant differences in age, incidence of hypertension and diabetes (*p* < 0.05). Patients with CSVD score 4 were the oldest [68.50 (P25, P75: 62.25, 74.75) years, *p* = 0.000] and had the highest incidence of hypertension (88.5%, *p* = 0.000). The incidence of diabetes in patients with CSVD score 3 was the highest (41.6%, *p* = 0.001).

**Table 1:** Comparison of clinical data by total CSVD burden score.

Clinical data	Total CSVD burden score						*p*-value
	No CSVD group (*n* = 82)	1-point group (*n* = 57)	2-point group (*n* = 59)	3-point group (*n* = 53)	4-point group (*n* = 52)	*χ*^2^ value	
Age (years)—median (IQR)	46.00 (33.75, 55.00)	58.00 (53.00, 63.00)	60.00 (54.00, 68.00)	65.00 (58.00, 72.50)	68.50 (62.25, 74.75)	93.368	0.000
Male, *n* (%)	49 (59.8%)	39 (68.4%)	39 (66.1%)	37 (69.8%)	41 (78.8%)	5.507	0.239
Smoking, *n* (%)	28 (34.1%)	19 (33.3%)	22 (37.3%)	19 (35.8%)	19 (35.8%)	0.288	0.991
Diabetes, *n* (%)	10 (12.2%)	13 (22.8%)	21 (35.6%)	22 (41.6%)	12 (23.1%)	18.211	0.001
Hyperlipidemia, *n* (%)	10 (12.2%)	8 (14.0%)	11 (18.6%)	5 (9.4%)	1 (1.9%)	8.233	0.083
Atrial fibrillation, *n* (%)	1 (1.2%)	0 (0%)	2 (3.4%)	2 (3.8%)	3 (5.8%)	4.564	0.335
Coronary heart disease, *n* (%)	2 (2.4%)	5 (8.8%)	5 (8.5%)	2 (3.8%)	6 (11.5%)	5.819	0.213
Hypertension, *n* (%)	18 (22.0%)	28 (49.1%)	43 (72.9%)	40 (75.5%)	46 (88.5%)	77.271	0.000

CSVD, cerebral small vessel disease; IQR, interquartile range.

### 3.2 Comparison of IAC grade between the five groups

IAC grades were significantly different five groups (*p* = 0.000), 82.9% of the patients with CSVD score of 0 did not have IAC. Patients with CSVD score of 1 have the highest incidence of IAC grade 1 (56.1%), patients with CSVD score of 2 have the highest incidence of IAC grade 2 (47.5%), patients with CSVD score of 3 have the highest incidence of IAC grade 3 (64.2%), and patients with CSVD score of 4 have the highest incidence of IAC grade 4 (46.2%), as shown in Table 2.

**Table 2:** Comparison of IAC grade by total CSVD burden score.

IAC evaluation	Total CSVD burden score					*χ*^2^ value	*p*-value
	No CSVD group (*n* = 82)	1-point group (*n* = 57)	2-point group (*n* = 59)	3-point group (*n* = 53)	4-point group (*n* = 52)		
No IAC, *n* (%)	68 (82.9%)	8 (14.0%)	3 (5.1%)	3 (5.7%)	0 (0%)	147.774	0.000
IAC grade 1, *n* (%)	7 (8.5%)	32 (56.1%)	9 (15.3%)	7 (13.2%)	10 (19.2%)		
IAC grade 2, *n* (%)	2 (2.4%)	3 (5.3%)	28 (47.5%)	5 (9.4%)	4 (7.7%)		
IAC grade 3, *n* (%)	3 (3.7%)	6 (10.5%)	11 (18.6%)	34 (64.2%)	14 (26.9%)		
IAC grade 4, *n* (%)	2 (2.4%)	8 (14.0%)	8 (13.6%)	4 (7.5%)	24 (46.2%)		

IAC, intracranial arterial calcification; CSVD, cerebral small vessel disease.

### 3.3 Incidence of other diseases in patients with EPVSs and patients with normal blood pressure

We found that EPVS is closely related to hypertension and brain atrophy, there were 288 patients with EPVS, Among EPVS patients, there are 171 cases (59.4%) with hypertension and 206 cases (68.0%) with brain atrophy. There were 146 cases (50.7%) with hypertension and brain atrophy.

In addition, we found some interesting results, IAC and aCSVD existed in 59 (46.1%) of 128 subjects with normal blood pressure.

### 3.4 Correlation analysis between aCSVD and IAC

Spearman rank correlation analysis showed that IAC was positively correlated with total CSVD burden score, deep WMHs, periventricular WMHs, CMBs, lacunes, and EPVSs, The results show that the more serious IAC is, the more serious aCSVD is as shown in Table 3.

**Table 3:** Correlation between single MRI markers of CSVD and IAC.

IAC	MRI markers of CSVD					
	Deep WMHs	Periventricular WMHs	CMBs	Lacunes	EPVS	Total CSVD burden score
*r_s_*	0.539	0.570	0.479	0.541	0.554	0.681
*p*-value	0.000	0.000	0.000	0.000	0.000	0.000

MRI, magnetic resonance imaging; CSVD, cerebral small vessel disease; IAC, intracranial arterial calcification; CMBs, cerebral microbleeds; EPVS, enlarged perivascular spaces; WMHs, white matter hyperintensities; *r_s_*, correlation coefficient.

Binary logistic regression analysis was carried out with aCSVD (present/absent) as the dependent variable. After adjusting for confounding factors (diabetes, hypertension, and age), the final model indicated aCSVD was independently associated with IAC grade 1–2 (OR = 23.747, 95% CI: 8.376–67.327; *p* = 0.000) and IAC grade 3–4 (OR = 30.166, 95% CI: 8.295–109.701; *p* = 0.000). This result shows that IAC is independently related to aCSVD, compared with people without IAC, patients with IAC grade 1–2 were 23.747 times more likely to suffer from aCSVD. Patients with IAC grade 3–4 were 30.166 times more likely to suffer from aCSVD, as shown in Table 4.

**Table 4:** Estimates from logistic regression analyses investigating the association between IAC and aCSVD.

Variable	Regression coefficient	Standard errors	Wald value	OR (95% CI)	*p*-value
IAC grade 1–2	3.167	0.532	35.490	23.747 (8.376, 67.327)	0.000
IAC grade 3–4	3.407	0.659	26.748	30.166 (8.295, 109.701)	0.000
Diabetes	−0.018	0.525	0.001	0.982 (0.351, 2.746)	0.972
Hypertension	1.700	0.452	14.155	5.471 (2.257, 13.262)	0.000
Age (≧52 years old)	1.290	0.499	6.678	3.632 (1.365, 9.659)	0.010

IAC, intracranial arterial calcification; aCSVD, arteriosclerotic cerebral small vessel disease; CI, confidence interval; OR, odds ratio.

Multiple-ordered logistic regression analysis was conducted with aCSVD severity (CSVD score 1–4) as the dependent variable. After adjusting for the confounding factors of diabetes, hypertension, and age, the final model indicated that IAC grades 3–4 were independently associated with aCSVD severity. Among patients with aCSVD, the probability of increasing aCSVD by 1 point was 4.697 times higher among those with IAC grade 3–4 than those without IAC, as shown in Table 5.

**Table 5:** Estimates from logistic regression analyses investigating the association between IAC and aCSVD severity.

Variable	Regression coefficient	Standard errors	Wald value	OR (95% CI)	*p*-value
IAC grade 1–2	0.244	0.604	0.163	1.276 (0.391, 4.170)	0.686
IAC grade 3–4	1.547	0.637	5.901	4.697 (1.349, 16.346)	0.015
Diabetes	−0.248	0.277	0.803	0.780 (0.453, 1.343)	0.370
Hypertension	1.190	0.288	17.021	3.287 (1.866, 5.783)	0.000
Age	0.038	0.013	8.813	1.039 (1.013, 1.064)	0.003

IAC, intracranial arterial calcification; aCSVD, arteriosclerotic cerebral small vessel disease; CI, confidence interval; OR, odds ratio.

## 4 Discussion

To our knowledge, this is the first study to combine MRI markers of CSVD into an overall measure of CSVD burden when investigating the association between aCSVD and IAC. In doing so, we determined that IAC may be a risk factor for aCSVD. Specifically, IAC severity was found to be positively correlated with WMHs, lacunes, CMBs, and EPVS. Additionally, IAC was correlated with total CSVD burden score.

### 4.1 The relationship between IAC and WMHs

Chung et al. (18) determined that IAC was positively correlated with deep WMHs (*r* = 0.417, *p* < 0.001) and periventricular WMHs (*r* = 0.388, *p* < 0.001). A study by Chen et al. (15) found that IAC was positively correlated with WMHs (*r* = 0.350, *p* < 0.001). The study also reported that IAC was positively correlated with deep WMHs (*r* = 0.503, *p* < 0.001) and periventricular WMHs (*r* = 0.535, *p* < 0.001). However, our *R*-value was slightly higher than their results, which may have been related to the methods used to evaluate calcification. Specifically, they used the methods proposed by Erbay (22) and Agatston (15), while our current study used the Babiarz method. Our previous study compared the Agatston, improved Woodcock, and Babiarz methods for evaluating calcification in patients with aCSVD and found that the Babiarz method was the best (23). Specifically, Babiarz method is determined to be simpler, faster and more accurate than the improved Woodcock and Agaston methods, and it will not cause less bias in the results, so it is more suitable for clinical work. It could also be used to retrospectively analyze existing CT data and evaluate the correlation between IAC and risk of various cerebrovascular diseases. Finally, unlike our current study, Tao et al. (24) reported that IAC was not related to WMHs. However, this difference in findings may be explained by the fact that Tao et al. only include patients with ischemic stroke and middle cerebral artery occlusion.

### 4.2 The relationship between IAC and EPVS

Chen et al. (15) did not find any significant associations between EPVS and IAC, while Tao et al. (24) found that greater EPVS and lower IAC were protective factors for IAC. Conversely, in a study of 437 patients, Del Brutto et al. (16) reported a significant correlation between EPVS and IAC, and determined that the probability of EPVS in patients with IAC grade 4 was three times higher than that in patients with IAC grade 1. The results of this current study show that IAC is positively correlated with EPVS. The perivascular space is an important brain waste clearance system. When head and neck atherosclerosis occurs, the wall of the brain artery thickens and hardens, limiting vasodilation and impairing fluid drainage and waste removal in the perivascular space. These changes also reduce oxygen and nutrition supply to the brain tissue, which may be one of the mechanisms of EPVS. Therefore, the IAC score may indirectly reflect some of the changes that occur in the perivascular space. Moreover, we found that 68.0% of patients with EPVS had brain atrophy. 50.7% of patients with EPVS has both hypertension and brain atrophy. The mechanism may be that EPVS is caused by the stretching of perivascular tissues after brain atrophy (25). while patients with hypertension, Increased intraluminal pressure may cause greater extravasation of fluid through the small arteries into perivascular spaces which is supported by rat experiments in which sustained hypertension could cause increased permeability of endothelial cells and fluid-induced damage to surrounding brain tissue (26). Moreover, elevated pulsatility in these areas could lead to enlargement of perivascular spaces because of the close proximity of EPVS to brain parenchyma (26).

### 4.3 The relationship between IAC and lacunes

This study found that IAC was positively associated with lacunes. Del Brutto et al. conducted two studies of Atahualpa residents in Ecuador with contradicting results. In their 2016 study, they reported that IAC was independently related to lacunes (OR = 3.1, 95% CI: 1.3–7.6; *p* = 0.013) (16), whereas their 2017 study did not find any relationship between IAC and lacunes (OR = 0.23, 95% CI = −0.12 to 0.58; *p* = 0.199) (17). These contradicting findings may be due to differences in calcification grade groupings. In the 2016 study, participants were divided into low and high calcification groups, whereas in the 2017 study they were divided into four groups (calcification grades 1–4).

### 4.4 The relationship between IAC and CMBs

A large study in Rotterdam found that after adjusting for confounding factors such as age, sex, cardiovascular disease, and ultrasonic carotid plaque score, there was no association between carotid artery calcification and CMBs in any of their three models (14). However, a Korean study involving 834 patients (27) found a strong correlation between IAC and deep CMBs (OR = 3.51, 95% CI: 2.39–5.14; *p* = 0.000). Lastly, similar to this current study, a study in China (15) determined that IAC was positively correlated with CMBs (*r* = 0.251, *p* < 0.05). Consistent with previous studies (9–11), this current study determined that age and hypertension are risk factors for CSVD. We presumed that the relationship between cerebral arterial calcification and hypertension could be synergistic or additive for the development of hypertensive brain damage. IAC leads to decreased arterial compliance, resulting in increased cerebral perfusion pressure and arterial wave energy, thus damaging cerebral microcirculation and leading to CMBs.

### 4.5 The relationship between IAC and total CSVD burden

This current study combined four neuroimaging markers of CSVD (lacunes, WMHs, CMBs, and EPVS) to study the relationship between total CSVD burden and IAC. The total CSVD burden score better reflects the overall impact of CSVD on brain tissue than only considering one or two markers alone. No other study has explored the relationship between total CSVD burden and IAC, so it is impossible to directly compare the results of our study to others. After adjusting for age, diabetes, and hypertension, we found evidence of a strong correlation between IAC and aCSVD, both when the presence/absence of CSVD and CSVD scores 1–4 were the dependent variables. Compared with people without IAC, patients with IAC grade 1–2 were about 23 times more likely to suffer from aCSVD. Compared with people without IAC, patients with IAC grade 3–4 were about 30 times more likely to suffer from aCSVD. Among patients with aCSVD, the probability of increasing aCSVD by 1 point was about 4.7 times higher among those with IAC grade 3–4 than those without IAC. Since this is a retrospective case-control study, we were unable to establish a causal relationship between aCSVD and IAC. However, when considering physiological pathology, IAC is more likely a risk factor for aCSVD, as the reverse seems unlikely. aCSVD is a type of microvascular disease. However, internal carotid artery calcification is a relatively large arterial lesion. This current study’s results indicate that there is an obvious correlation between arterial calcification and aCSVD. We propose three possible research directions in the future, We hypothesize that there are three possible explanations for how these lesions lead to aCSVD. First, IAC leads to aortic lumen stenosis, which in turn leads to chronic hypoperfusion and brain tissue injury (28, 29). Second, laminar flow is obstructed by arterial stenosis. Turbulence and wall shear force changes occur at the distal end of the stenosis, causing vascular endothelial cell damage and microthrombosis, which results in perforating artery embolism in the brain and lacunar cerebral infarction. At the same time, vascular endothelial cell damage leads to the destruction of the blood–brain barrier and WMHs. Moreover, increases in matrix metalloproteinase-2 lead to the expansion of the space around the perforating artery and the formation of EPVSs (12, 30). Third, IAC leads to decreased arterial compliance, resulting in increased cerebral perfusion pressure and arterial wave energy, thus damaging cerebral microcirculation and leading to WMHs, lacunes, and CMBs (31). According to these three conjectures, we can further study the pathogenesis of aCSVD, and study the influence on CSVD from the macrovascular lesion, which is a new perspective.

### 4.6 The relationship between CSVD and IAC and other risk factors

Our research results show that 17.1% of patients without-CSVD remaining with some degree of IAC, and 8.3% of patients with 1–3-point CSVD load score, Have no-IAC. The pathogenesis of CSVD is complex, and CSVD is not caused by a single factor. Besides IAC found in our research, we also found that age and hypertension are risk factors for aCSVD, which is similar to previous reports (9–11). In addition, inflammatory factor and the destruction of blood–brain barrier are the key factors of CSVD (32–34).

Long-term chronic hypertension is considered as the main risk factor for IAC and aCSVD. However, our data showed that IAC and aCSVD existed in 59 (46.1%) of 128 subjects with normal blood pressure. Arterial calcification is considered to be an integral part of the active process of atherosclerosis, which occurs in up to 90% of atherosclerotic lesions. Arterial calcification is an important sign of advanced atherosclerotic lesions. However, the risk factors of atherosclerotic lesions are not only hypertension, but also obesity, smoking, Psychosocial stress, Sleep deprivation, ambient air polarization, smoking, Passive exposure to tobacco smoke and so on (35). A large study in Sweden shows that high salt intake is an important risk factor for atherosclerosis even without hypertension (36). The result of a study in China shows that high normal blood pressure is related with higher risk of Carotid Artery Atherosclerosis in this cohort (37). But, a small increase in blood pressure within a physiological range has a substantial impact on plaque development. Earlier intervention in elevated blood pressure may prevent or delay morbidity associated with atherosclerosis (38). Therefore, for patients with aCSVD without hypertension, it is still necessary to pay attention to the control of blood pressure and carry out early intervention to avoid further aggravating aCSVD.

## 5 Limitations

This study has some limitations. First, it was a case-control study of brain CT and MRI findings. Thus, enrolment of patients into the study may have introduced bias. In order to control the selection bias, the sample sizes of the five groups of subjects we selected were similar, and the subjects were TIA and AIS patients. In order not to affect the evaluation of CSVD, we excluded a large area of AIS patients. The selection bias would probably lead to an underestimation of the association between the total CSVD burden score and IAC. Second, since this is a case-control study, we could only establish correlations between IAC and aCSVD. A large, longitudinal study is needed to establish a causal relationship between IAC and aCSVD. Third, the total CSVD burden score may vary among the study population, and our cases are all from China population, our results might not be generalizable to other populations or ethnic groups and further collaborative studies are required to corroborate our findings.

## 6 Conclusion

In conclusion, IAC is associated with the total CSVD burden score and single imaging signs. aCSVD is a disease caused by microvascular disease in the brain. The results of this study show that there is also a correlation between aCSVD and IAC, which provides a new viewpoint for the etiology explanation of aCSVD and a new direction for the in-depth study of its pathogenesis.

## Data availability statement

The raw data supporting the conclusions of this article will be made available by the authors, without undue reservation.

## Ethics statement

The studies involving humans were approved by the Ethics Committee of Guangxi International Zhuang Medicine Hospital. The studies were conducted in accordance with the local legislation and institutional requirements. Written informed consent for participation in this study was provided by the participants’ legal guardians/next of kin.

## Author contributions

PC: Funding acquisition, Supervision, Writing – original draft, Writing – review & editing. TiL: Supervision, Writing – review & editing. YW: Supervision, Writing – review & editing. ZM: Data curation, Writing – review & editing, Writing – original draft. TaL: Data curation, Writing – original draft, Writing – review & editing. SL: Data curation, Writing – original draft, Writing – review & editing. JX: Data curation, Writing – original draft, Writing – review & editing. SM: Data curation, Writing – original draft, Writing – review & editing.
